# An Efficient Deep Learning Approach for Malaria Parasite Detection in Microscopic Images

**DOI:** 10.3390/diagnostics14232738

**Published:** 2024-12-05

**Authors:** Sorio Boit, Rajvardhan Patil

**Affiliations:** College of Computing, Grand Valley State University, Grand Rapids, MI 49503, USA

**Keywords:** malaria, diagnosis, deep learning

## Abstract

**Background:** Malaria is a life-threatening disease spread by infected mosquitoes, affecting both humans and animals. Its symptoms range from mild to severe, including fever, muscle discomfort, coma, and kidney failure. Accurate diagnosis is crucial but challenging, relying on expert technicians to examine blood smears under a microscope. Conventional methods are inefficient, while machine learning approaches struggle with complex tasks and require extensive feature engineering. Deep learning, however, excels in complex tasks and automatic feature extraction. **Objective:** This paper presents EDRI, which is a novel hybrid deep learning model that integrates multiple architectures for malaria detection from red blood cell images. The EDRI model is designed to capture diverse features and leverage multi-scale analysis. **Methods:** The proposed EDRI model is trained and evaluated on the NIH Malaria dataset comprising 27,558 labeled microscopic red blood cell images. **Results:** Experiments demonstrate its effectiveness, achieving an accuracy of 97.68% in detecting malaria, making it a valuable tool for clinicians and public health professionals. **Conclusions:** The results demonstrate the effectiveness of proposed model’s ability to detect malaria parasite from red blood cell images, offering a robust tool for rapid and reliable malaria diagnosis.

## 1. Introduction

Malaria remains one of the most pressing global health challenges especially in low- and middle-income countries where it contributes significantly to morbidity and mortality [1]. According to the World Health Organization’s 2021 report, there were an estimated 241 million malaria cases worldwide, leading to approximately 627,000 deaths, predominantly among children under five in sub-Saharan Africa [1]. The effective management and control of malaria heavily rely on timely and accurate diagnosis to ensure prompt treatment and reduce transmission [2]. Traditional diagnostic methods primarily involve the microscopic examination of Giemsa-stained blood smears, which is considered the gold standard due to its ability to detect and quantify different Plasmodium species [3]. However, this method is labor-intensive, time consuming, and requires skilled personnel, limiting its utility in resource-poor settings [4]. Rapid diagnostic tests (RDTs), which detect specific antigens produced by malaria parasites, offer a quicker alternative but suffer from reduced sensitivity and specificity, particularly at low parasite densities and with non-falciparum species [5,6]. These limitations underscore the necessity for more efficient, accurate, and automated diagnostic approaches. Advancements in artificial intelligence (AI) and deep learning have opened new avenues in medical diagnostics, offering automated analysis with high accuracy and efficiency [7,8]. Convolutional Neural Networks (CNNs), a class of deep learning models, have shown promise in image recognition tasks, including the detection of malaria parasites in microscopic images [9]. Despite these developments, existing AI-based methods for malaria detection face challenges such as high computational demands, limited generalizability across diverse datasets, and insufficient interpretability [10]. Many state-of-the-art models are computationally intensive, hindering their deployment in settings with limited access to advanced hardware. Moreover, variability in staining techniques, imaging conditions, and morphological differences across datasets can affect the models’ ability to generalize and accurately classify parasitized cells [11]. To address these challenges, we propose a novel hybrid deep learning model named EDRI (EfficientNetB2-Dense-Residual-Inception). The EDRI model integrates the EfficientNetB2 architecture [12] with DenseNet [13], ResNet [14], and Inception [15] blocks to form a robust network capable of extracting and processing diverse features from microscopic RBC images. The EfficientNetB2 backbone provides efficient feature extraction through compound scaling, while the Dense and Residual blocks enhance feature propagation and reuse. The Inception modules facilitate multi-scale analysis, allowing the model to capture features at various levels of abstraction.

There are five main contributions of this work:

1. Propose the development of the EDRI model, an efficient deep learning architecture that can accurately detect malaria from red blood cell images while maintaining computational efficiency.
2. We evaluate the EDRI model using the NIH Malaria dataset [8], using performance metrics such as accuracy, precision, recall, F1 score, and AUC, compared to existing methods.
3. We validate the EDRI model’s design choices involves conducting an ablation study that systematically examines the impact of its constituent components on overall performance.
4. Extensive experiments are also conducted using baseline models, providing a rigorous benchmarking of the EDRI model’s efficacy against established deep learning architectures.
5. Discussion of practical implications, including the model’s suitability for deployment in resource-limited settings and its potential integration into mobile health platforms and IoT systems for remote diagnostics

The remainder of this paper is organized as follows: Section 2 reviews related work on malaria detection methods and AI applications in medical diagnostics. Section 3 details the proposed EDRI model, including its architectural components and design rationale. Section 4 describes the dataset and preprocessing techniques used in this study. Section 5 outlines the experimental setup, including hardware configurations, evaluation metrics, and training protocols. Section 6 presents and discusses the results of the model evaluation. Finally, Section 7 concludes the paper and suggests directions for future research.

## 2. Background and Related Work

### 2.1. Traditional Malaria Detection Methods

#### 2.1.1. Microscopic Examination

The microscopic examination of Giemsa-stained blood smears remains the gold standard for malaria diagnosis due to its ability to detect and differentiate between Plasmodium species and stages [1,2]. This method involves the manual inspection of blood smears by trained technicians to identify the presence of malaria parasites. While it is highly specific and allows for parasite quantification, it is labor-intensive, time consuming, and requires significant expertise [3,4]. The accuracy of this method is highly dependent on the skill level of the technician and the quality of the microscope, leading to variability in diagnostic performance, especially in resource-limited settings where experienced personnel and equipment may be scarce [5].

#### 2.1.2. Antigen-Based Rapid Diagnostic Tests (RDTs)

Antigen-based RDTs have been developed to provide quick and user-friendly diagnostic alternatives by detecting specific parasite antigens in a patient’s blood sample, typically yielding results within 15–30 min [6,7]. These tests are particularly useful in remote areas lacking microscopy facilities. However, RDTs have limitations in sensitivity and specificity, especially in detecting low parasitemia levels common in asymptomatic individuals or in infections with non-falciparum species [8,9]. False negatives can occur in patients with low parasite loads, and false positives may result from cross-reactivity with other infections or residual antigens from previous infections [10]. Moreover, the storage conditions and shelf life of RDTs can affect their performance, posing challenges in tropical environments [11].

#### 2.1.3. Limitations of Traditional Approaches

Despite their historical significance and widespread use, traditional malaria diagnostic methods face several critical limitations that impede effective disease management, particularly in resource-constrained settings. One of the primary challenges is the dependence on skilled personnel. Microscopic examination requires highly trained technicians who can accurately identify malaria parasites among blood cells [12]. In many endemic regions, there is a scarcity of such skilled healthcare workers, creating a significant bottleneck in the diagnostic process and limiting access to reliable malaria testing. Moreover, the procedures involved in manual microscopy are inherently time consuming and labor-intensive [13]. Preparing high-quality blood smears, staining them correctly, and thoroughly examining them under a microscope demand considerable time and meticulous effort. This lengthy process is not conducive to high-throughput screening, leading to delays in diagnosis and treatment. In situations where prompt medical intervention is crucial, such delays can result in increased morbidity and mortality. Variability and human error further compromise the effectiveness of traditional diagnostic methods. The accuracy of microscopic diagnosis can vary significantly due to factors such as technician fatigue, differences in expertise, and subjective interpretation of visual findings [14]. These inconsistencies can lead to misdiagnosis, either by overlooking parasitic infections or by false identification, thereby affecting patient outcomes and potentially contributing to the spread of the disease. Additionally, antigen-based rapid diagnostic tests (RDTs), although providing quicker results, suffer from sensitivity issues. They may not detect low-level parasitemia, which is common in asymptomatic carriers [15]. These individuals, despite not exhibiting symptoms, can still transmit the parasite to others. The inability of RDTs to identify such cases poses a significant challenge to malaria control and eradication efforts, as it hinders the detection of all infection reservoirs within a population. Collectively, these limitations underscore the urgent need for automated, accurate, and efficient diagnostic tools that can function effectively in resource-limited environments. Advancements in technology, particularly in artificial intelligence and machine learning, offer promising avenues to overcome these challenges. Such innovations have the potential to transform malaria diagnostics and greatly improve global health outcomes through reduced reliance on skilled personnel, minimized human error, and enhanced sensitivity to low-level infections.

### 2.2. Machine Learning and Deep Learning for Malaria Detection

#### 2.2.1. Advancements in Machine Learning Techniques

The advent of machine learning and deep learning, particularly Convolutional Neural Networks (CNNs), has significantly transformed image-based diagnostics by automating feature extraction and classification tasks [16,17]. In the field of malaria detection, CNNs have demonstrated exceptional accuracy in distinguishing between parasitized and uninfected red blood cells (RBCs) from microscopic images [18,19]. These models learn hierarchical features directly from raw pixel data, capturing complex patterns and subtle differences that may be challenging for human observers to discern. Consequently, CNNs have emerged as powerful tools in enhancing the accuracy and efficiency of malaria diagnosis.

#### 2.2.2. CNN-Based Malaria Detection Models

Several studies have explored the application of CNNs and their variants to improve malaria diagnosis. For example, Quan et al. [20] proposed the Attentive Dense Circular Network (ADCN) model which combines the DenseNet architecture with attention mechanisms to enhance feature representation. The ADCN achieved an accuracy of 97.47% on the NIH Malaria dataset by leveraging attention modules that focus on the most relevant features within microscopic images. This result demonstrated the effectiveness of attention modules in enhancing model performance, enabling the network to zero in on critical areas that indicate malaria infection. In a different approach, Umer et al. [21] introduced a Stacked CNN architecture that attained an accuracy of 99.98%. By stacking multiple convolutional layers, the model captures hierarchical features at various levels of abstraction, thereby improving classification performance. This architecture underscores the potential of deeper networks in capturing intricate patterns associated with parasitized cells. Similarly, Goni et al. [22] developed a CNN model incorporating a Parasite Inflator mechanism, which is referred to as CNN-DELM. This innovative approach amplifies parasite features within the images, addressing the challenge of detecting parasites in low-contrast or noisy environments. The CNN-DELM model achieved an accuracy of 99.66%, demonstrating the efficacy of enhancing feature representation to improve detection rates. Further advancements include the work of Musaev et al. [23], who proposed an ICNN-Ensemble model. This ensemble of CNNs operates on high-resolution image channels and combines predictions from multiple networks to improve robustness and generalization. The ICNN-Ensemble achieved an accuracy of 99.67%, illustrating the benefits of ensemble methods in reducing variance and improving predictive performance in malaria detection tasks. Another notable contribution is from Pamungkas et al. [24], who leveraged the EfficientNet-B0 model, which is known for its efficient scaling of network parameters. By optimizing depth, width, and resolution, EfficientNet-B0 balances performance and computational efficiency. Their implementation achieved an accuracy of 97.37%, showcasing the applicability of EfficientNet architectures in resource-constrained settings without significant loss of accuracy. Moreover, Dev et al. [25] explored the combination of CNNs with Recurrent Neural Networks (RNNs), including Long Short-Term Memory (LSTM) and Gated Recurrent Unit (GRU) layers, to capture both spatial and temporal features in RBC images. Their hybrid CNN-RNN model achieved an accuracy of 96.20%, demonstrating the potential of integrating different types of neural networks to enhance feature extraction and capture sequential dependencies within image data. These studies collectively highlight the rapid evolution of deep learning applications in malaria detection. Researchers have explored diverse architectures, including attention mechanisms, stacked layers, ensemble models, and hybrid networks, to progressively refine diagnostic accuracy. These advancements not only improve performance but also address practical challenges such as computational efficiency and robustness to variations in image quality.

#### 2.2.3. Advanced Network Architectures

Researchers have explored advanced network architectures to further enhance feature extraction and classification performance in malaria detection tasks. Two notable architectures, DenseNet and ResNet, have been instrumental in addressing the vanishing gradient problem prevalent in deep neural networks [26,27]. DenseNet introduces dense connections between layers, ensuring maximum information flow by connecting each layer to every other subsequent layer in a feed-forward fashion [26]. This design promotes feature reuse and alleviates the vanishing gradient issue, enabling the training of deeper networks. Similarly, ResNet employs residual connections that allow layers to learn residual functions with reference to the layer inputs, effectively simplifying the learning process for very deep networks [27]. In addition to these architectures, Inception modules have been adopted to capture multi-scale features within images [28]. The Inception architecture processes input data through multiple convolutional filters of different sizes in parallel, enabling the network to learn both fine-grained and coarse features simultaneously. Dong et al. [19] implemented Inception modules in their model for malaria detection, achieving an accuracy of 97.5%, thereby demonstrating the effectiveness of multi-scale feature extraction in improving classification outcomes.

#### 2.2.4. Challenges and Limitations of Existing Models

Despite the impressive accuracies reported by various deep learning models, several challenges hinder their practical deployment in malaria detection. Models trained on specific datasets often struggle to perform well on images from different laboratories or field conditions due to variations in staining techniques, imaging equipment, and sample preparation methods [29,30]. This lack of robustness limits the applicability of these models in diverse real-world settings where consistent image quality cannot be guaranteed. Furthermore, high-performance models typically require substantial computational resources for both training and inference, including high-end GPUs or specialized hardware [31]. This requirement poses a significant barrier to deployment in resource-limited settings or on mobile devices, where computational capabilities are constrained. Additionally, the scarcity of larger annotated datasets necessitates the effective training of deep learning models, which can lead to overfitting and poor generalization to new samples [32]. This data scarcity is particularly challenging for rare diseases or in regions with limited infrastructure for data collection and annotation.

### 2.3. Our Proposed EDRI Model

We introduce the EDRI model, which is an innovative hybrid convolutional neural network designed to overcome existing limitations by integrating multiple advanced architectures. Building on the EfficientNetB2 backbone, which utilizes compound scaling to optimize depth, width, and resolution for enhanced accuracy with fewer parameters [33], the EDRI model ensures computational efficiency suitable for resource-constrained environments. The incorporation of DenseNet and ResNet blocks facilitates effective gradient flow and feature reuse, allowing the network to learn complex patterns and mitigate the vanishing gradient problem [26,27]. Additionally, the inclusion of Inception modules enables multi-scale feature extraction by applying parallel convolutional filters of varying sizes, thereby improving the model’s ability to generalize across diverse imaging conditions and accurately detect malaria parasites [28]. This comprehensive integration results in a robust and efficient solution for malaria detection.

## 3. Methodology

### 3.1. Integrating EfficientNetB2, DenseNet, ResNet, and Inception Blocks

The EDRI model, a novel hybrid CNN, addresses the research gap for efficient malaria detection from microscopic RBC images [18]. Designed for accurate and efficient malaria detection from microscopic RBC images, this innovative model leverages the strengths of diverse architectural blocks to achieve comprehensive feature extraction and enhanced classification accuracy. EfficientNetB2 serves as the backbone, providing a strong yet computationally efficient feature extraction capability through compound scaling [33]. Dense blocks are utilized to enrich feature representation by concatenating outputs from all preceding layers, promoting feature reuse and improving the learning of intricate patterns [26]. Residual blocks address the vanishing gradient problem, enabling the effective training of deeper networks by allowing layers to learn residual functions with reference to the layer inputs [27]. Inception blocks facilitate multi-scale feature extraction by applying convolutional filters of different sizes in parallel, capturing complex spatial information essential for distinguishing between parasitized and uninfected cells [28]. This hybrid approach leverages the individual strengths of each architectural component to create a model that is both accurate and suitable for deployment in resource-constrained environments.

The EDRI model’s hybrid architecture leverages the advantages of multiple convolutional neural network blocks, including EfficientNetB2, DenseNet, ResNet, and Inception blocks. This combination enables the model to learn hierarchical features, intricate patterns, address the vanishing gradient problem, and capture complex spatial information by combining spatial and contextual information across multiple scales using concatenation and residual connections. The EfficientNetB2 backbone extracts a feature map with 2048 channels, which is enriched through Dense, Residual, and Inception blocks. These blocks enhance feature reuse, stability, and multi-scale analysis through skip connections and concatenation. The final output after the Inception block is reduced to 256 features via Global Average Pooling, which is further processed by a Dense layer with 512 neurons for robust classification.

### 3.2. Architectural Design

**Figure 1:**
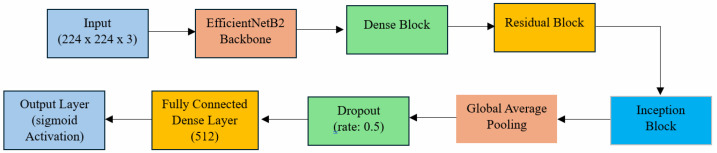
Architecture of proposed EDRI model.

The EDRI model is composed of interconnected layers that synergistically enhance feature extraction and classification performance. The architecture begins with the EfficientNetB2 backbone, excluding its fully connected layers to allow for integration with custom modules tailored to the specific task of malaria detection. Following the base layers, Dense blocks are incorporated to build upon the extracted features. By concatenating multiple convolutional outputs, Dense blocks create rich feature maps that provide a detailed understanding of the underlying patterns within the RBC images [26]. This extensive cross-layer connectivity enhances feature propagation and mitigates the vanishing gradient problem. The architecture integrates Residual blocks for the stable training of deeper models. Residual connections facilitate gradient flow, improving convergence and enabling the network to learn complex features effectively [27]. This design also adapts to different feature scales, mitigates the vanishing gradient problem, and strikes a balance between depth and performance, leading to state-of-the-art results. With this design, the network can learn rich representations and generalize well to new data. Additionally, the Residual blocks enable efficient training and inference, making the model scalable and versatile. Figure 1 illustrates the architecture of the proposed EDRI model, which integrates EfficientNetB2 as the backbone, which is followed by Dense, Residual, and Inception blocks. This combination and interconnection between components enhances feature extraction, contributing to improved classification performance.

The introduction of Inception blocks enables the network to capture multi-scale features through the parallel processing of inputs using convolutional filters of varying sizes. This allows for the analysis of features at different scales, enhancing the network’s ability to learn diverse spatial features from input images [28]. The significance of this capability is heightened in malaria parasite detection, where size and shape variations are pronounced. The network’s ability to recognize and adapt to these variations through Inception blocks is important for accurate detection. Building on the strengths of Residual blocks, Inception blocks, and Dense blocks, the network is designed to effectively extract features and learn complex patterns. Following the convolutional layers, Global Average Pooling is employed to reduce feature map dimensions and prevent overfitting [34]. This is followed by a Dropout layer, which randomly drops units during training, encouraging the network to develop robust features and further mitigating overfitting [35]. Additionally, the final classification layer employs a sigmoid activation function, outputting probabilities for the two classes (Parasitized and Uninfected), making it ideal for binary classification tasks where a single threshold value determines class membership. Finally, a fully connected Dense layer with sigmoid activation is used to predict the binary outcome of parasitized or uninfected cells.

### 3.3. Advantages over Existing Approaches

The EDRI model’s hybrid design provides distinct advantages over conventional deep learning models used in malaria detection. By integrating EfficientNetB2, Dense, Residual, and Inception blocks, the model achieves enhanced feature extraction capabilities, enabling more precise differentiation between parasitized and uninfected cells [26,27,28,33]. This integration not only improves the granularity of feature detection but also promotes robustness against variations in input data, which is critical for handling diverse imaging conditions encountered in field diagnostics. The EfficientNetB2 backbone ensures computational efficiency, which is pivotal for deploying the model in settings with limited hardware capabilities. Furthermore, the combination of Dense and Residual blocks facilitates deeper network architectures without suffering from the vanishing gradient problem, thereby maintaining high accuracy and stability during training [26,27]. Inception blocks contribute to the model’s ability to perform multi-scale analysis, which is crucial for adapting to various sizes and shapes of malaria parasites as they appear in microscopic images [28]. Collectively, these features make the EDRI model not only more accurate but also more generalizable and efficient compared to existing approaches, underscoring its suitability for real-world applications in resource-constrained settings.

## 4. Materials and Methods

### Dataset and Data Preprocessing

This study utilized the NIH Malaria dataset, which is publicly available and consists of 27,558 microscopic images of red blood cells (RBCs) [36]. These images, derived from Giemsa-stained thin blood smear slides, represent samples from 150 malaria-infected and 50 healthy individuals, ensuring a balanced representation of parasitized and uninfected cells. The dataset includes parasitized RBCs, showcasing a variety of morphological changes associated with different stages of malaria infection. In contrast, uninfected samples may contain non-parasitic artifacts like staining interferences or dust, which introduce realistic variability into the dataset [18,37]. For preprocessing, all images were resized to 224 × 224 pixels to meet the input size requirements of EfficientNetB2, optimizing computational efficiency without losing crucial details [33]. The normalization of pixel values to a range of 0 to 1 ensures uniform input scales, aiding in faster convergence during model training [38].

**Figure 2:**
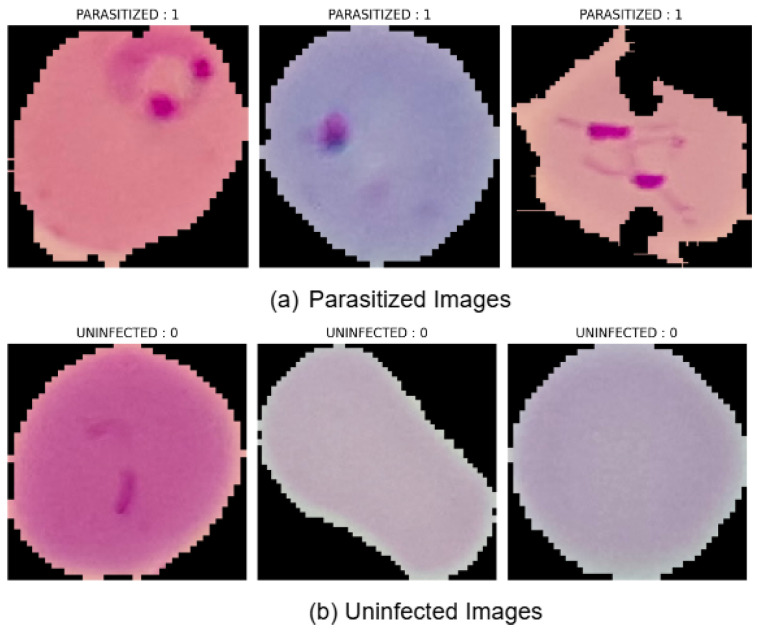
Sample images from the red blood cell dataset, showing both parasitized and uninfected cells.

Additionally, data augmentation techniques such as random rotations, shifts, zooming, shearing, and flipping were applied to enhance the dataset’s diversity and the model’s ability to generalize across varied imaging conditions [32,39]. The dataset was divided into training (80%), validation (10%), and testing (10%) subsets through stratified sampling, ensuring balanced distribution and the representation of both classes in each subset [40]. This approach ensured that the model was trained and evaluated on representative data, minimizing bias and improving its ability to generalize to unseen images. Figure 2 shows an example image from the NIH Malaria dataset, depicting a parasitized and uninfected red blood cell. This visual provides an understanding of the type of microscopic images used to train and evaluate the model.

## 5. Experimental Setup

### 5.1. Hardware and Software Configuration

Experiments were conducted on the Google Cloud Platform (GCP), leveraging high-performance virtual machines with advanced GPUs and TPUs. This setup enabled efficient scaling and accelerated processing for extensive model training and evaluation tasks. The software environment comprised Python 3.8 and TensorFlow 2.x as the deep learning framework along with essential libraries such as Keras, NumPy, and Pandas for data manipulation and model development [41].

### 5.2. Training Protocol

The EDRI model was trained using the Adam optimizer [42], which was selected for its adaptive learning rate capabilities that are well suited for deep neural networks. Training proceeded over 50 epochs with a batch size of 32, striking a balance between computational efficiency and convergence speed. To mitigate overfitting and optimize training progress, the model employed an early stopping strategy, halting training after five consecutive epochs without validation loss improvement [43]. Additionally, the ReduceLROnPlateau callback adjusted the learning rate downwards by a factor of 0.2 whenever the validation loss did not decrease for three consecutive epochs, ensuring continued learning during later training stages [43]. The model’s weights were initially set with the EfficientNetB2 backbone partially frozen, utilizing pre-trained ImageNet weights to enhance early learning stability [33,44]. Table 1 includes the summary of training protocol used for the proposed model.

In the initial training phase, the EfficientNetB2 backbone was partially frozen to leverage the pre-trained weights from ImageNet. Specifically, the bottom layers of the network were frozen, while the top 40 layers were unfrozen and trainable. This partial freezing enabled the model to retain generic feature extraction capabilities learned from ImageNet while focusing on optimizing higher-level features specific to malaria parasite detection. This configuration kept only the top layers trainable at the start. After 10 epochs, the network was completely unfrozen, allowing for fine tuning across all layers. This gradual unfreezing approach helped the model adapt more effectively to the specific characteristics of the malaria dataset, enhancing its generalization capabilities and further preventing overfitting. Furthermore, we did not apply k-fold cross-validation, since the dataset was sufficiently large and well distributed. Also, stratified splits ensured balanced training, validation, and test sets, mitigating the risk of data bias. Also, given the balanced and extensive nature of the NIH Malaria dataset, stratified splitting provided a robust evaluation framework, minimizing computational overhead while ensuring fair representation across subsets.

### 5.3. Performance Metrics

In this study, the evaluation of model performance was conducted using various metrics, including precision, recall, F1 score, and accuracy. Following the training phase, the model’s effectiveness and classification capabilities were assessed using a separate testing dataset. Additionally, the confusion matrix was employed to evaluate performance, providing insights into true positives (TP), true negatives (TN), false positives (FP), and false negatives (FN). Before presenting the results, below, we define these evaluation metrics.

Accuracy: Accuracy is the proportion of correct predictions (both true positives and true negatives) among all predictions. It is computed as shown below in Equation (1).

(1) Accuracy=(TP+TN)/(TP+TN+FP+FN)

where

1. TP: The model’s true positive rate indicates the proportion of actual positive instances correctly predicted as positive.
2. TN: The true negative rate represents the correct identification of negative cases by the model, out of all negative instances.
3. FP: The false positive rate highlights the instances where the model mistakenly classifies negative cases as positive.
4. FN: The false negative rate reveals the instances where the model incorrectly predicts positive cases as negative.
5. Accuracy: This metric calculates the model’s overall correctness, determined by dividing the sum of correct predictions (TP + TN) by the total number of predictions made.

Precision: Precision represents the model’s ability to minimize false positives and is defined as Equation (2).

(2) Precision=TP/(TP+FP)

Precision is particularly important in medical diagnostics, where a high precision reduces the risk of false positives, ensuring that cells predicted as parasitized are indeed infected.

Recall: Recall measures the model’s ability to identify all actual positives (parasitized cells), which are calculated as shown below in Equation (3).

(3) Recall=TP/(TP+FN)

A high recall value indicates that the model is effective in detecting parasitized cells, which is essential for minimizing missed diagnoses.

F1 score: The F1 score is the harmonic mean of precision and recall, providing a balanced measure between the two, especially when the data are imbalanced [45]. It is computed as shown below in Equation (4).

(4) F1Score=(Precision∗Recall)/(Precision+Recall)

This metric is critical in healthcare settings, balancing the trade-off between false positives and false negatives.

AUC-ROC: The Area Under the Receiver Operating Characteristic Curve (AUC-ROC) is used to evaluate the model’s ability to distinguish between parasitized and uninfected cells across all classification thresholds [46]. It measures the area under the ROC curve, where AUC is computed using Equation (5).

(5) $$AUC=\int_{0}^{1}TPR\left(t\right)dFPR\left(t\right)$$

The TPR (true positive rate) is equivalent to recall, and the FPR (false positive rate) is calculated as shown in Equation (6).

(6) FPR=FP/(FP+TN)

## 6. Results and Discussion

The EDRI model was evaluated on the test set of the NIH Malaria dataset, achieving an overall accuracy of 97.68%. This level of accuracy indicates that the model correctly classified a vast majority of the images, effectively distinguishing between parasitized and uninfected red blood cells (RBCs). The model’s precision of 98.88% reflects its ability to minimize false positives, ensuring that most cells identified as parasitized were indeed infected. Similarly, the recall of 96.44% demonstrates the model’s effectiveness in identifying actual parasitized cells, reducing the incidence of false negatives. The F1 score of 97.65%, being the harmonic mean of precision and recall, signifies a strong balance between the two, which is crucial in medical diagnostics where both false positives and false negatives can have serious implications.

The Area Under the Receiver Operating Characteristic Curve (AUC-ROC) of 99.76% further highlights the model’s exceptional discriminative ability across all classification thresholds. An AUC close to 1.0 indicates that the model performs almost perfectly in distinguishing between the two classes. Additionally, the log loss of 0.07 signifies that the model’s predictions are not only accurate but also confident with low uncertainty associated with its probabilistic outputs. Table 2 summarizes the performance of the proposed EDRI model.

These results demonstrate the model’s effectiveness in accurately identifying malaria-infected cells while maintaining a high level of robustness. The high precision indicates the reliable identification of parasitized cells without excessive false positives, while the high recall reflects the model’s capability to detect actual cases of infection effectively. The F1 score signifies a strong balance between precision and recall. An AUC-ROC close to 1.0 indicates excellent discriminative ability, and the low Log Loss value signifies confident and well-calibrated predictions.

### 6.1. Ablation Studies

The ablation study presented in Table 3 demonstrates that integrating Residual, Dense, and Inception modules with the EfficientNetB2 backbone significantly enhances model performance across all evaluated metrics. The proposed EDRI model, which combines all three modules, achieves better results with an accuracy of 97.68%, precision of 98.88%, recall of 96.44%, F1 score of 97.65%, AUC of 99.76%, and the lowest loss of 0.07. Models omitting any one of these modules exhibit diminished performance, underscoring the essential contribution of each component to the network’s efficacy. In contrast, the baseline EfficientNetB2 model attains a lower accuracy of 95.00% and a higher loss of 0.24, highlighting the substantial improvements provided by the integrated modules. This analysis confirms that the synergistic combination of Residual, Dense, and Inception architectures markedly enhances predictive capabilities and overall model performance.

### 6.2. Baseline Comparison Results

Table 4 presents the results of experiments conducted using various pre-trained convolutional neural network architectures for malaria detection, revealing considerable variations in their performance. Among the standard models, DenseNet121 achieved the highest accuracy of 94.30% and an AUC of 98.38%, outperforming VGG16, VGG19, and NASNetMobile, which exhibited lower accuracies and higher loss values. InceptionV3 and Xception also demonstrated strong performance with accuracies of 93.80% and 94.05%, respectively. The EfficientNet series showed mixed results. EfficientNetB1 stood out with a 96.00% accuracy and a higher AUC of 99.64%, while EfficientNetB0 and EfficientNetB2 had lower accuracies and higher losses. Notably, the proposed EDRI model surpassed all baseline architectures, attaining an accuracy of 97.68%, precision of 98.88%, recall of 96.44%, F1 score of 97.65%, AUC of 99.76%, and the lowest loss of 0.07. These comprehensive experiments underscore the efficacy of the EDRI model in accurately detecting malaria, highlighting its potential as a highly effective tool for clinical diagnostics.

### 6.3. Testing Accuracy and Loss Curves

**Figure 3:**
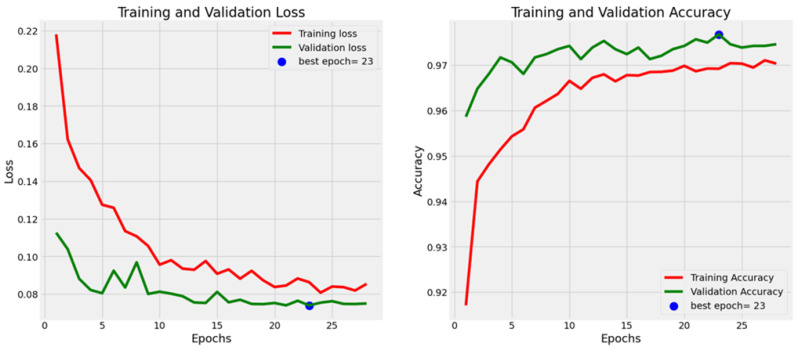
Loss values and accuracy of proposed models during the training and validation.

The training and validation loss and accuracy curves Figure 3 provide insights into the performance of the proposed model and other fine-tuned deep learning models over the training period. The graphs depict how the models evolved with the proposed model showing a consistent reduction in loss and a steady increase in accuracy with each epoch. In the loss curves, a rapid decline in training loss is observed, indicating efficient learning from the onset. The validation loss follows a similar downward trend, reflecting the model’s ability to generalize well to new data without substantial overfitting. This trend signifies that the model is learning effectively while maintaining a balance between fitting the training data and generalizing to unseen samples. The accuracy curves further emphasize the model’s strong performance, as evidenced by high accuracy with minimal errors on validation data. Notably, the proposed model outperforms the alternative models with validation accuracy reaching a plateau above 97%—a clear indicator of its reliability in distinguishing between parasitized and uninfected cells. Optimal performance was achieved around epoch 23, which was marked by stable, high accuracy and minimal fluctuations, particularly from epoch 15 onward. These smooth and stable curves highlight the model’s robustness, showcasing its capacity to learn effectively without overfitting, making it a reliable solution for malaria detection tasks.

### 6.4. Confusion Matrix

We assessed the model’s performance by testing it on a separate validation dataset, which is distinct from the training dataset. This evaluation phase allowed us to examine the model’s ability to generalize and make accurate predictions on new, unseen data, which is crucial for real-world applicability.

**Figure 4:**
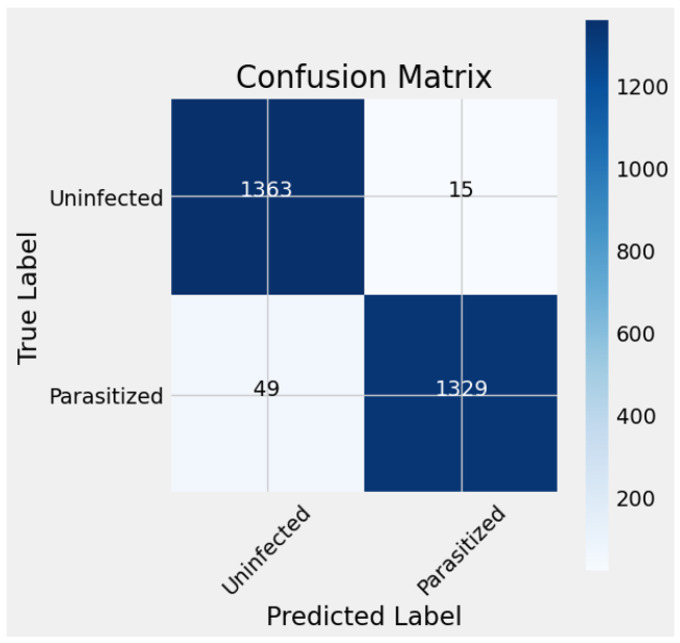
Confusion matrices of the proposed model.

The model generated predictions based on the validation dataset, providing insights into its effectiveness and robustness. The confusion matrix in Figure 4 summarizes the model’s performance, revealing a high number of true negatives (1363) and true positives (1329), indicating the model’s reliability in distinguishing between parasitized and uninfected samples. The low counts for false positives (15) and false negatives (49) demonstrate the model’s ability to minimize incorrect classifications, reducing unnecessary concern or intervention. The mis-classifications could stem from image artifacts such as staining interference, blurred focus, or low-contrast regions, which are inherently challenging for both machine and human interpretation. Overall, the proposed model demonstrates a strong capacity to generalize to new samples with the confusion matrix confirming its effectiveness in identifying malaria-infected cells with minimal errors, highlighting its potential for reliable malaria detection.

**Figure 5:**
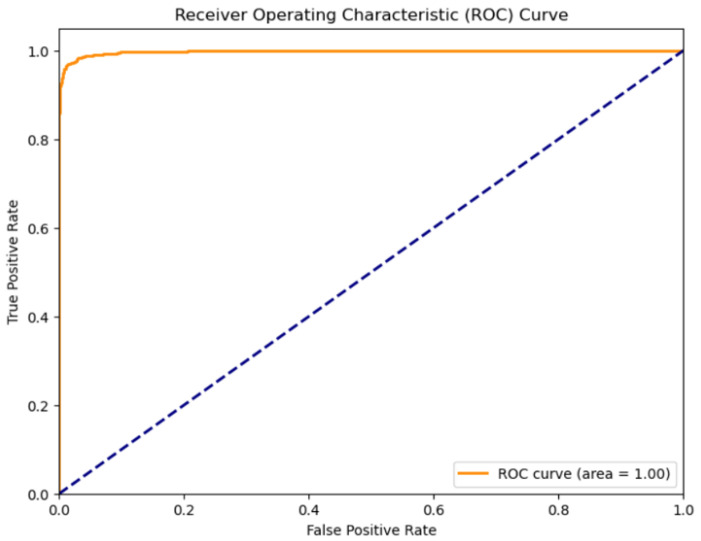
AUROC curve of the proposed model.

Additionally, the ROC curve in Figure 5 visually represents the model’s ability to distinguish between classes by plotting the true positive rate (TPR) against the false positive rate (FPR). With an Area under the Curve (AUC) value of 1.00, the model demonstrates discriminatory power, achieving near-perfect classification performance. The ROC curve serves as a valuable metric in classification tasks, providing insights into the model’s sensitivity and specificity across various threshold levels.

### 6.5. Comparative Analysis with Existing Models

In contextualizing the performance of the EDRI model, a comparative analysis was conducted against existing studies utilizing the NIH Malaria dataset, focusing on key evaluation metrics such as accuracy, precision, recall, F1 score, and AUC. Table 5 showcases the result of this comparative analysis. Kumar et al. (2017) [47] applied a fine-tuned Convolutional Neural Network (CNN), achieving a 95% accuracy; however, the absence of additional performance metrics limited the depth of comparison. Similarly, Dong et al. (2017) [19] reported a slightly higher accuracy of 95.28% with their CNN architecture but also did not detail other crucial indicators. Rajaraman et al. (2018) [48] enhanced performance to a 95.90% accuracy and F1 score through an ensemble of pre-trained CNN models, but this approach increased computational complexity. In contrast, Vijayalakshmi and Kanna (2019) [49] utilized a VGG-19 model combined with a Support Vector Machine (SVM) classifier, attaining 93% accuracy and a 91% F1 score on a smaller dataset, which may affect the generalizability of their results. Bibin et al. (2017) [50] achieved a higher accuracy of 97.37% and a sensitivity (recall) of 96.58% using Deep Belief Networks (DBNs), yet they did not report precision, F1 score, or AUC values necessary for comprehensive evaluation. Liang et al. [18] reached a 96.54% accuracy and 96.70% recall with their CNN-based system but similarly lacked additional metric reporting. Hemachandran et al. (2020) [51] demonstrated that MobileNetV2 outperformed other architectures with a 97.06% accuracy and a 96.77% AUC, highlighting its effectiveness and computational efficiency for mobile deployment. In comparison, the EDRI model surpassed these studies by achieving an accuracy of 97.68%, a precision of 98.88%, a recall of 96.44%, an F1 score of 97.65%, and an impressive AUC of 99.76%. These metrics indicate not only higher accuracy but also better performance across all critical evaluation criteria, including low false positives and high true positive rates. Furthermore, the EDRI model’s hybrid architecture, which integrates EfficientNetB2 with Dense, Residual, and Inception blocks, facilitates efficient feature extraction, improved gradient flow, and multi-scale feature representation, enhancing generalization across diverse imaging conditions essential for clinical applications. Additionally, the model’s computational efficiency makes it suitable for deployment in resource-constrained environments, unlike more complex ensemble methods or deeper architectures that demand extensive computational resources. While previous studies have achieved notable results with the NIH Malaria dataset, the EDRI model distinguishes itself by delivering high performance across multiple evaluation metrics while maintaining computational efficiency. This balance of accuracy, robustness, and efficiency positions the EDRI model as a promising tool for real-world malaria detection, particularly in resource-limited settings where such solutions are critically needed.

### 6.6. Implications for Practical Deployment

The high performance of the EDRI model demonstrates its potential for practical application in malaria-endemic regions. Its ability to accurately and efficiently detect malaria parasites in microscopic images can aid in rapid diagnosis, facilitating timely treatment and reducing the burden on healthcare systems. The model’s computational efficiency makes it suitable for integration into mobile devices or point-of-care diagnostic tools, expanding access to reliable malaria testing in remote or under-resourced areas. Furthermore, the use of a publicly available dataset and open-source tools ensures that the EDRI model can be reproduced and adapted by other researchers and practitioners. This accessibility promotes collaboration and continuous improvement, which are essential for addressing global health challenges such as malaria.

### 6.7. Limitations of the Study

Despite the promising results, certain limitations must be acknowledged. The model was trained and evaluated on a single dataset, the NIH Malaria dataset [36], which, while comprehensive, may not capture the full diversity of imaging conditions and parasite variations encountered in different geographic regions. Variations in microscope equipment, staining protocols, and local parasite species could affect the model’s performance when applied to new data sources. Additionally, the dataset comprises images with a balanced distribution of parasitized and uninfected cells, which may not reflect the actual prevalence rates in clinical settings, where uninfected cells are often more prevalent. This discrepancy could impact the model’s performance in real-world applications, potentially requiring further calibration or retraining with more representative datasets.

## 7. Conclusions

This study introduces EDRI, which is an innovative and efficient hybrid convolutional neural network for malaria parasite detection from red blood cell images. The EDRI model marks a significant leap forward in the application of deep learning for malaria detection. By leveraging a hybrid architecture combining EfficientNetB2 with Dense, Residual, and Inception blocks, the model has achieved exemplary performance metrics [19]. Traditional malaria detection methods often lack efficiency, exhibit lower accuracy, and require extensive computational resources. In contrast, EDRI achieved an accuracy of 97.8% and an AUC score of 99.76%, highlighting its robust performance. These results demonstrate the model’s capability to contribute meaningfully to clinical and field diagnostics, offering a robust tool for rapid and reliable malaria diagnosis. Its high accuracy, precision, recall, and AUC-ROC highlight its potential to alleviate the global burden of malaria. In the future, we aim to expand and diversify the training dataset to capture a broader range of geographical and demographic variations, which will aid in increasing the model’s generalizability [48]. We plan to develop lightweight versions of EDRI for deployment on mobile and IoT devices, enabling use in resource-limited settings to support healthcare professionals with timely and accurate malaria diagnoses. Additionally, future work will explore multi-class classification to identify different Plasmodium species and infection stages as well as integrate explainable AI techniques to foster trust and interpretability in clinical settings. Furthermore, we also plan to investigate advanced deep learning techniques such as attention mechanisms or generative adversarial networks, which could potentially heighten the model’s diagnostic accuracy and robustness [49]. Implementing real-world clinical trials will be crucial to verify the model’s practical performance and ensure its adaptability to varied clinical settings [50].

## Figures and Tables

**Table 1:** Summary of training protocol.

Parameters	Value
Optimizer	Adam [42]
Initial Learning Rate	0.0001
Training Epochs	50
Batch Size	32
Early Stopping	Triggered after 5 epochs
Learning Rate Adjustment	Factor of 0.2 reduction after 3 epochs
Model Weights Initialization	EfficientNetB2 backbone partially frozen

**Table 2:** Performance of proposed model on the test set.

Metric	Value
Accuracy	97.68%
Precision	98.88%
Recall	96.44%
F1 Score	97.65%
AUC-ROC	99.76%
Log Loss	0.07

**Table 3:** Ablation studies of model performance.

Model Version	Accuracy	Precision	Recall	F1 Score	AUC	Loss
EfficientNetB2 + Residual + Inception (No Dense)	95.97%	98.90%	92.96%	95.85%	99.43%	0.11
EfficientNetB2 + Dense + Inception (No Residual)	96.37%	97.90%	94.78%	96.31%	99.15%	0.12
EfficientNetB2 + Dense + Residual (No Inception)	97.27%	98.01%	96.47%	97.25%	99.46%	0.08
EfficientNetB2 Backbone Only	95.00%	95.91%	94.00%	94.94%	98.60%	0.24
Proposed EDRI model	97.68%	98.88%	96.44%	97.65%	99.76%	0.07

**Table 4:** Performance metrics of pre-trained models for malaria detection.

Model	Accuracy	Precision	Recall	F1 Score	AUC	Loss
VGG16	91.55%	91.83%	91.55%	91.53%	96.84%	0.22
VGG19	89.26%	89.39%	89.26%	89.25%	94.75%	0.29
InceptionV3	93.80%	93.83%	93.80%	93.79%	98.14%	0.17
DenseNet121	94.30%	94.37%	94.30%	94.30%	98.38%	0.16
MobileNetV2	93.11%	93.27%	93.11%	93.10%	98.11%	0.18
Xception	94.05%	94.13%	94.05%	94.05%	98.02%	0.17
NASNetMobile	84.66%	78.88%	94.66%	86.06%	96.14%	0.35
EfficientNetB0	93.00%	95.74%	90.00%	92.78%	95.04%	0.27
EfficientNetB1	96.00%	94.23%	98.00%	96.08%	99.64%	0.12
EfficientNetB2	95.00%	95.92%	94.00%	94.95%	98.60%	0.24
EfficientNetB3	89.00%	84.21%	96.00%	89.72%	96.88%	0.35
Proposed Model	97.68%	98.88%	96.44%	97.65%	99.76%	0.07

**Table 5:** Performance metrics of pre-trained models for malaria detection in NIH Malaria dataset.

Reference	Method	No. of Images	Accuracy (%)	Precision (%)	Recall (%)	F1 Score (%)	AUC (%)
Dong et al. (2017) [19]	CNN	27,558	95.28	95.10	95.50	–	–
Rajaraman et al. (2018) [48]	Ensemble of pre-trained CNNs	27,558	95.90	–	–	95.90	–
Vijayalakshmi and Kanna (2019) [49]	VGG-19 + SVM	2550	93.00	89.95	93.44	91.66	–
Bibin et al. (2017) [50]	Deep Belief Network	27,558	97.37	–	96.58	–	–
Liang et al. [18]	CNN	27,558	96.54	–	96.70	–	–
Hemachandran et al. (2020) [51]	MobileNetV2	27,558	97.06	97.00	97.00	98.00	96.77
Dong et al. (2017) [19]	CNN	27,558	95.28	95.10	95.50	–	–
Rajaraman et al. (2018) [48]	Ensemble of Pre-trained CNNs	27,558	95.90	–	–	95.90	–
Proposed EDRI Model	EfficientNetB2 + Dense, Residual, Inception Blocks	27,558	97.68	98.88	96.44	97.65	99.76

## Data Availability

The original data presented in the study are openly available at https://lhncbc.nlm.nih.gov/LHC-research/LHC-projects/image-processing/malaria-datasheet.html (accessed on 1 July 2024).
